# The impact of depression among chronic low back pain patients in Japan

**DOI:** 10.1186/s12891-016-1304-4

**Published:** 2016-10-27

**Authors:** Toshinaga Tsuji, Ko Matsudaira, Hiroki Sato, Jeffrey Vietri

**Affiliations:** 1 Medical Affairs Department, Shionogi & Co., Ltd., Osaka, Japan; 2 Department of Medical Research and Management for Musculoskeletal Pain, 22nd Century Medical and Research Center, The University of Tokyo, Tokyo, Japan; 3 Health Outcomes Practice, Kantar Health, 700 Dresher Road, Horsham, PA 19044 USA

**Keywords:** Low back pain, Chronic low back pain, Depression, Quality of life

## Abstract

**Background:**

Chronic low back pain (CLBP) is associated with significant disability and reductions in health related quality of life (HRQoL), which can negatively impact overall function and productivity. Depression is also associated with painful physical symptoms, and is often present in patients with chronic pain. However, the incremental burden associated with depression or symptoms of depression among CLBP patients is not well understood. The objective of this study was to investigate the impact of depression on HRQoL in CLBP and to assess the relationship between depression and work impairment and healthcare use among CLBP patients in Japan.

**Methods:**

Data were extracted from the 2014 Japan National Health and Wellness Survey (*N* = 30,000). CLBP was defined by report of diagnosed low back pain ≥3 months duration. Depression was assessed using the Patient Health Questionnaire (PHQ-9). Measurements assessed included pain, HRQoL, labor force participation, work productivity and healthcare utilization. Patients with depression (PHQ-9 ≥ 10) were compared to patients without depression (PHQ-9 < 10) using t-tests for continuous and count variables and chi-square for categorical variables, which were followed by generalized linear models adjusted for covariates. The association between presenteeism and other patient outcomes and characteristics was analysed using nonparametric correlations (Spearman’s rho).

**Results:**

Depressed CLBP patients had significantly more severe pain and higher levels of pain compared with patients without depression (*P* < 0.001). Depression was associated with worse HRQoL in CLBP patients. Presenteeism, overall work impairment and activity impairment were 1.8, 1.9 and 1.7 times as high, respectively, among those with depression relative to those without depression. CLBP patients with depression had almost twice as many healthcare provider visits in 6 months than those without depression. The pattern of results remained consistent after adjustment for sociodemographic and general health characteristics. Analysis also indicated presenteeism was closely related to overall work impairment (rho = 0.99).

**Conclusions:**

Depression among CLBP patients in Japan was associated with higher pain scores and lower HRQoL scores, as well as lower labor productivity and increased healthcare use. Screening for depression in CLBP patients should be an essential part of CLBP patient care.

## Background

Low back pain (LBP) is a common health issue affecting at least 80 % of individuals during their lifetime [1] and poses a severe economic burden on individuals and their communities [2–5]. The Global Burden of Disease Study 2013 found that globally, back pain was one of the leading cause of years lived with disability (YLDs) [6]. In Japan, back pain is the top cause of YLD and the 2^nd^ and 4^th^ most frequent reason for outpatient visits for women and men, respectively [6, 7].

One of the main characteristics of LBP is recurrence, and a number of patients develop chronic LBP (CLBP). In Japan CLBP is the most prevalent type of chronic pain [8], with a prevalence estimated at 23 %, and 11–12 % of the population is disabled by it [9]. Though considerable research has been directed at understanding back pain, most Japanese epidemiological studies examine LBP in general, with few focused on CLBP [10–12].

While burdensome in its own right, pain is also risk factor for depression, and many studies have examined the co-occurrence of pain and depression [13–16]. The comorbidity is clinically well established but the underlying mechanisms are not well understood, though a potential explanation is disruption of the mesolimbic dopamine system [17, 18]. Recent data from animal models indicate that regulation of dopamine activity in the ventral tegmental area (VTA) mediates depressive and anxiogenic responses [19] suggesting a neurological link between depression and chronic pain.

CLBP in particular is often co-morbid with depression [20], a main cause of disability worldwide [6]. Depression increases the risk of developing LBP [21], and CLBP is affected by the patient’s mental state [22]. In spite of that, the mental state of most CLBP patients is not routinely assessed. Thus, in chronic pain, psychosocial risk factors become relevant, and are important to explain how individuals respond to back pain. Recent studies have demonstrated that psychosocial factors are important risk factors for LBP among Japanese workers [22, 23]; however, data examining the role of depression in CLBP patients in Japan is lacking.

The objective of this study was to investigate the impact of depression on health-related quality of life (HRQoL) in CLBP, as well as to assess the relationship between depression and work impairment and healthcare use among CLBP patients in Japan.

## Methods

### Sample

Data were extracted from the 2014 Japan National Health and Wellness Survey (NHWS) (Kantar Health, New York, USA), which is a general health survey designed to reflect the health of the population in Japan (*N* = 30,000). The survey is administered via the Internet, with potential respondents identified through opt-in survey panels. Participants were stratified by gender and age groups to ensure representative samples, with quotas set through the distribution of age and gender within the Japanese population aged ≥18 years.

Respondents were considered to have CLBP if they had been diagnosed with back pain by a doctor, reported experiencing back pain in the past month, and experienced back pain ≥ 3 months. Three months duration of LBP is considered chronic according to both Japanese and US treatment guidelines [24, 25]. Depression symptoms and severity of depression over the last two weeks was assessed using the Patient Health Questionnaire (PHQ-9), a validated scale used to screen for depression and assess its severity [26]. The scale evaluates depression by measuring the frequency of anhedonia, depressed mood, sleep disturbance, lack of energy, appetite disturbance, negative self-feelings, difficulty concentrating, psychomotor retardation or agitation, and thoughts of self-harm. A single-item measure of the interference of these symptoms was also included. Respondents who scored ≥ 10 (the cutoff associated with moderate depression) were considered to have depression regardless of whether they indicated a diagnosis of depression, and respondents scoring <10 (associated with minimal or mild depression) were considered not to have depression; this value has shown good sensitivity and specificity for major depression in previous research [27].

### Measures

Using a 0–10 numeric rating scale (NRS) anchored by *No Pain* (0) and *Pain as Bad as You Can Imagine* (10), respondents rated the severity of their LBP, as well as the severity of their pain overall, as mild (0–3), moderate (4–6), or severe (7–10). The NRS was completed for both current and pain in the past week. Respondents indicated how frequently they experienced problems with pain on a 6-point scale ranging from *Daily* to *Once a month or less often*. HRQoL was measured using the revised Medical Outcomes Study 36-Item Short Form Survey Instrument (SF-36v2;[28]). This is a multipurpose, generic HRQoL instrument comprising 36 questions. The instrument is designed to report on eight health concepts (physical functioning (PF), role physical (RP), bodily pain (BP), general health (GH), vitality (VT), social functioning (SF), role emotional (RE), and mental health (MH)). The versions of the scores used in this study were based on the Japanese norms, which have a mean of 50 and standard deviation of 10 in the Japanese population [29]. Scores can be interpreted relative to this population average of 50 as well as with other comparison groups of interest. Higher scores indicate better quality of life.

Mental component summary (MCS), physical component summary (PCS), and short form 6D (SF-6D) health utility scores were also calculated according to the standard scoring algorithms. These scores are based on the US (MCS & PCS) and UK (SF-6D) general populations, but are commonly reported in studies outside those countries as the scores allow for comparison across international populations.

Labor force participation was defined as being employed or unemployed but looking for work. Work productivity was assessed using the Work Productivity and Activity Impairment (WPAI) questionnaire, a 6-item validated instrument which consists of four metrics: absenteeism (the percentage of work time missed because of one's health in the past seven days), presenteeism (the percentage of impairment experienced while at work in the past seven days because of one's health), overall work productivity loss (an overall impairment estimate that is a combination of absenteeism and presenteeism), and activity impairment (the percentage of impairment in daily activities because of one's health in the past seven days) [30]. Only respondents who reported being employed full-time or part-time provided data for absenteeism, presenteeism, and overall work impairment. All respondents provided data for activity impairment.

Healthcare utilization was defined by the number of healthcare provider visits, the number of hospital emergency room (ER) visits, and the number of times hospitalized in the past six months. The reason for each visit was not included in the questionnaire.

### Analysis

The analysis was primarily concerned with the association between the presence of depression, so patients with depression (PHQ-9 ≥ 10) were compared with those without depression (PHQ-9 < 10) using t-tests for continuous and count variables and chi-square for categorical variables. To ensure differences due to confounding variables were not attributed to depression, these tests were followed by regression modelling using generalized linear models adjusting for age, sex, length of LBP diagnosis, Charlson Comorbidity Index (CCI), household income, marital status, university education, body mass index (BMI), cigarette smoking, alcohol use, and exercise to account for sociodemographic characteristics and general health characteristics.

These comparisons according to were supplemented by correlational analysis, using the PHQ-9 score as a continuous measure. Because some outcomes were positively skewed rather than normally distributed, the association between presenteeism and other patient outcomes and characteristics was analysed using nonparametric correlations (Spearman’s rho).

## Results

Of the participants surveyed, 425 were identified as having CLBP. The average age of a respondent with CLBP was 54 years old, and 44 % were female (Table 1). When assessed according to depression status, CLBP patients with depression (PHQ-9 ≥ 10; *N* = 70) were younger than CLBP patients without depression (PHQ-9 < 10; *N* = 355) by approximately 9 years on average, but did not differ in terms of average CCI score, gender, or employment status. Patients with depression were less likely to be married or live with a partner (Table 1). Patients indicated their LBP was either mild (47 %) or moderate (44 %) rather than severe (9 %). Both overall severity of pain and current level of pain were near the midpoint of the NRS, and almost half reported daily problems with pain. Depression was significantly associated with more severe pain and higher levels of pain, current and in the prior week (Table 1).

**Table 1:** Characteristics of CLBP patients according to presence of depression

	Total (*N* = 425)	Depression (PHQ-9 ≥ 10) (*N* = 70)	No Depression (PHQ-9 < 10) (*N* = 355)	*P value*
Age, Mean ± SD	53.90 ± 14.16	45.91 ± 13.73	55.48 ± 13.73	<0.001
Female, n (%)	187 (44.00)	33 (47.14)	154 (43.38)	0.562
Employment status, n (%)				0.589
Not currently employed	164 (38.59)	25 (35.71)	139 (39.15)	
Employed	261 (61.41)	45 (64.29)	216 (60.85)	
Annual household income, n (%)				0.079
< ¥3million	83 (19.53)	22 (31.43)	61 (17.18)	
¥3million to < ¥5million	100 (23.53)	15 (21.43)	85 (23.94)	
¥5million to < ¥8million	113 (26.59)	15 (21.43)	98 (27.61)	
¥8million or more	97 (22.82)	12 (17.14)	85 (23.94)	
Decline to answer	32 (7.53)	6 (8.57)	26 (7.32)	
Marital status, n (%)				0.021
Single/Divorced/Separated/Widowed	138 (32.47)	31 (44.29)	107 (30.14)	
Married/living with partner	287 (67.53)	39 (55.71)	248 (69.86)	
Education level, n (%)				0.063
Less than university education	218 (51.29)	43 (61.43)	175 (49.30)	
University education or higher	207 (48.71)	27 (38.57)	180 (50.70)	
Body mass index category, n (%)				0.137
Underweight	52 (12.24)	9 (12.86)	43 (12.11)	
Normal weight	280 (65.88)	39 (55.71)	241 (67.89)	
Overweight	70 (16.47)	16 (22.86)	54 (15.21)	
Obese	19 (4.47 %)	4 (5.71)	15 (4.23)	
Decline to provide weight	4 (0.94 %)	2 (2.86)	2 (0.56)	
Smoking behavior, n (%)				0.088
Never smoked	182 (42.82)	34 (48.57)	148 (41.69)	
Former smoker	132 (31.06)	14 (20.00)	118 (33.24)	
Current smoker	111 (26.12)	22 (31.43)	89 (25.07)	
Alcohol use, n (%)				0.975
Do not drink	116 (27.29)	19 (27.14)	97 (27.32)	
Drink alcohol	309 (72.71)	51 (72.86)	258 (72.68)	
Vigorous exercise at least one day in the past month, n (%)				0.198
Do not exercise	213 (50.12)	40 (57.14)	173 (48.73)	
Exercise	212 (49.88)	30 (42.86)	182 (51.27)	
Charlson comorbidity index, Mean ± SD	0.51 ± 2.23	0.83 ± 3.64	0.44 ± 1.83	0.186
Sleep difficulties, n (%)	82 (19.29)	35 (50.00)	47 (13.24)	<0.001
Severity of LBP, n (%)				<0.001
Mild	186 (47.45)	19 (29.23)	167 (51.07)	
Moderate	172 (43.88)	34 (52.31)	138 (42.20)	
Severe	34 (8.67)	12 (18.46)	22 (6.73)	
Missing	33	5	28	
Severity of pain in the prior week (0–10), Mean ± SD	4.48 ± 2.31	5.80 ± 2.26	4.23 ± 2.23	<0.001
Current severity of pain (0–10), Mean ± SD	4.59 ± 2.28	5.86 ± 2.27	4.34 ± 2.20	<0.001
Frequency of problems with pain, n (%)				0.002
Daily	188 (44.24)	44 (62.86)	144 (40.56)	
4–6 times a week	63 (14.82)	12 (17.14)	51 (14.37)	
2–3 times a week	82 (19.29)	10 (14.29)	72 (20.28)	
Once a week	39 (9.18)	3 (4.29)	36 (10.14)	
2–3 times a month	35 (8.24)	0 (0.00)	35 (9.86)	
Once a month or less often	18 (4.24)	1 (1.43)	17 (4.79)	
Type of diagnosing doctor for LBP, n (%)				0.028
General internist	18 (4.24)	4 (5.71)	14 (3.94)	
Gynecologist	5 (1.18)	0 (0.00)	5 (1.41)	
Orthopedist	353 (83.06)	54 (77.14)	299 (84.23)	
Rheumatologist	4 (0.94)	3 (4.29)	1 (0.28)	
Pain management specialist	3 (0.71)	1 (1.43)	2 (0.56)	
Other	42 (9.88)	8 (11.43)	34 (9.58)	
Type of prescribing doctor, n (%)				0.150
General internist	28 (16.87)	6 (15.38)	22 (17.32)	
Gynecologist	2 (1.20)	0 (0.00)	2 (1.57)	
Orthopedist	116 (69.88)	24 (61.54)	92 (72.44)	
Rheumatologist	5 (3.01)	3 (7.69)	2 (1.57)	
Pain management specialist	1 (0.60)	0 (0.00)	1 (0.79)	
Other	14 (8.43)	6 (15.38)	8 (6.30)	
Missing	259	31	228	
Duration of LBP (months), Mean ± SD	112 ± 120	96 ± 99	115 ± 123	0.227
Current use of a prescription medication for pain, n (%)				0.002
No	259 (60.94)	31 (44.29)	228 (64.23)	
Yes	166 (39.06)	39 (55.71)	127 (35.77)	
Current use of NSAIDs prescription for pain, n (%)				0.049
No	40 (24.10)	14 (35.90)	26 (20.47)	
Yes	126 (75.90)	25 (64.10)	101 (79.53)	
Missing	259	31	228	
Use of an OTC product for pain, n (%)				0.861
No	306 (72.00)	51 (72.86)	255 (71.83)	
Yes	119 (28.00)	19 (27.14)	100 (28.17)	
Use of an herbal product for pain, n (%)				0.441
No	413 (97.18)	69 (98.57)	344 (96.90)	
Yes	12 (2.82)	1 (1.43)	11 (3.10)	

^a^NSAIDS are prescription drugs in Japan. *CLBP* chronic low back pain, *LBP* low back pain, *NSAIDs* non-steroidal anti-inflammatory drugs, *OTC* over-the-counter, *PHQ*-*9* Patient Health Questionaire-9

CLBP patients with depression had worse HRQoL than CLBP patients without depression (Table 2). Depression was also associated with more impairment while at work (presenteeism). Overall work impairment, which is largely driven by presenteeism, was also significantly higher among CLBP patients with depression. There was no significant difference in absenteeism or rate of labor force participation between CLBP patients with and without depression. Depressed CLBP patients reported more activity impairment than those without depression. Depression was also associated with approximately two more healthcare provider visits among CLBP patients in the 6 month recall period (Table 2).

**Table 2:** Outcomes among CLBP patients according to presence of depression

	Total (*N* = 425)	Depression (PHQ-9 ≥ 10) (*N* = 70)	No Depression (PHQ-9 < 10) (*N* = 355)	
	Mean ± SD	Mean ± SD	Mean ± SD	*P* value
Health status: Japanese norm-based scores				
Physical functioning	44.36 ± 15.43	37.73 ± 17.9	45.66 ± 14.57	<0.001
Role physical	42.26 ± 14.29	32.51 ± 16.28	44.19 ± 13.05	<0.001
Bodily pain	39.59 ± 8.89	34.61 ± 9.56	40.57 ± 8.43	<0.001
General health	42.59 ± 10.96	33.25 ± 9.24	44.43 ± 10.32	<0.001
Vitality	42.87 ± 10.92	30.98 ± 9.26	45.22 ± 9.63	<0.001
Social functioning	43.03 ± 13.36	30.51 ± 13.63	45.49 ± 11.85	<0.001
Role emotional	44.7 ± 13.14	32.34 ± 15.13	47.14 ± 11.22	<0.001
Mental health	45.17 ± 11.2	32 ± 9.54	47.77 ± 9.55	<0.001
Health status: International scores				
Mental component	45.01 ± 10.92	31.27 ± 10.04	47.72 ± 8.85	<0.001
Physical component	46.81 ± 7.65	44.08 ± 7.96	47.35 ± 7.48	0.001
Health utility (SF-6D)	0.67 ± 0.12	0.56 ± 0.09	0.69 ± 0.11	<0.001
Work impairment				
Absenteeism %	4.92 ± 17.87	7.33 ± 22.37	4.39 ± 16.75	0.335
Presenteeism %	31.59 ± 28.08	46.43 ± 26.12	28.43 ± 27.52	<0.001
Overall work impairment %	33.90 ± 30.08	49.81 ± 27.74	30.40 ± 29.50	<0.001
Activity impairment %	37.34 ± 29.90	56.00 ± 27.21	33.66 ± 29.05	<0.001
HCP visits (past 6 months)	12.64 ± 16.24	19.67 ± 21.07	11.25 ± 14.75	<0.001

*HCP* healthcare provider

**Fig. 1:**
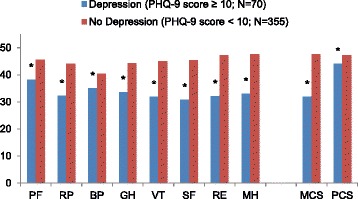
Adjusted mean HRQoL scores among CLBP patients according to presence of depression. *p < 0.05

The pattern of results was consistent after covariates were incorporated into the regression analysis. Adjusted HRQoL scores were lower on all of the eight Japanese norm-based scores. Adjusted mean MCS and PCS using international norms were also lower (Fig. 1).

**Fig. 2:**
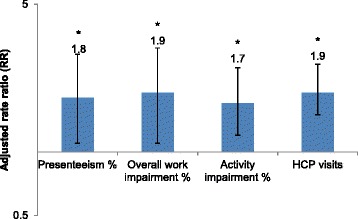
Adjusted impairments and healthcare visit rates among depressed CLBP patients relative to those without depression. *p < 0.05. Results are presented on a logarithmic scale; values above 1 (x-axis) indicate increased impairment and resource use among CLBP patients with depression

Regression-adjusted presenteeism and overall work impairment were 1.8 and 1.9 times as high, respectively, among those with depression relative to those without depression (Fig. 2). Activity impairment was 1.7 times as high in patients with depression compared with patients without depression after adjustment for covariates. HCP visits were almost twice as frequent in patients with depression compared with patients without depression. Likewise, work impairment was greater in patients with depression compared with patients without depression.

Analysis of depression based on PHQ-9 scores as a continuous variable also demonstrated the association between depression and pain among CLBP patients. Greater depression was significantly associated with more frequent problems with pain, greater current and past-week severity of pain (based on NRS scores), pain at more sites in addition to LBP, and more presenteeism and overall work impairment (*P* < 0.001, Table 3). Moreover, additional regression analysis conducted using PHQ-9 scores as a continuous variable corroborated the findings, indicating lower HRQoL scores with higher PHQ-9 scores, with the exception of the Japanese PCS score. Pain was likewise worse with greater depression as was presenteeism, overall work impairment, and activity impairment. Consistent with the results shown in Fig. 2, HCP visits were more frequent with greater depression scores, but there was no significant association with ER visits or hospitalizations (data not shown).

**Table 3:** Correlations between depression, pain, and work impairment among CLBP patients

	Spearman’s rho with PHQ-9 score^a^	*P* value
Frequency of problems with pain	0.236	<0.001
Current severity of pain (based on NRS score)	0.289	<0.001
Severity of pain in the prior week (based on NRS score)	0.332	<0.001
Additional pain sites (number, 0–6)	0.387	<0.001
Presenteeism %	0.340	<0.001
Overall work impairment %	0.342	<0.001

^a^Correlation is significant at the 0.01 level (2-tailed)

*CLBP* chronic low back pain, *NRS* numeric rating scale, *PHQ*-*9* Patient Health Questionnaire

When assessing the relationship between work impairment and other characteristic and outcomes, presenteeism was very closely related to overall work impairment (rho = 0.99). Greater presenteeism was associated with more-severe LBP, more-severe pain in the prior week and currently based on the NRS. Although, there was a trend for greater presenteeism being associated with more frequent problems with pain, it did not reach statistical significance (*P* = 0.08). Presenteeism was moderately related to the severity of depression according to the PHQ-9 score (Table 4).

**Table 4:** Correlations between presenteeism, pain, and depression among employed CLBP patients

	Spearman's rho with presenteeism	*P* value
Overall work impairment %	0.990^a^	<0.001
Severity of lower back pain	0.267^a^	<0.001
Severity of pain in the prior week (based on NRS)	0.297^a^	<0.001
Current severity of pain (based on NRS)	0.245^a^	<0.001
Frequency of problems with pain	0.115	0.077
Sites of pain in addition to LBP (number, 0–6)	0.239^a^	0.002
Depression severity based on PHQ-9	0.342^a^	<0.001

^a^Correlation is significant at the 0.01 level (2-tailed)

## Discussion

Our results demonstrated that CLBP patients with depression had significantly more severe and higher levels of pain, as well as significantly worse HRQoL compared with CLBP patients without depression. These observations are consistent with those recently published by Hiyama et al., which showed that depressed patients and those with neuropathic LBP had a higher level of pain and poorer quality of life compared with non-depressed patients [16]. The majority of patients had mild (47 %) or moderate (44 %) LBP. Current and prior week pain severity scores were similar (4.6/10 vs 4.5/10) and almost half of all patients reported daily pain problems. Overall sociodemographic patient characteristics were similar between the two groups of CLBP patients with the exception of age, marital status and sleeping difficulties. CLBP patients with depression were significantly younger, on average 9 years, compared with CLBP patients without depression. These observations tend to be consistent with observations for major depressive disorder where estimates in the general population are 15–17 %, while the 1-year prevalence rate in individuals ≥ 65 years is lower, at 1–4 % [31]. Significantly more CLBP patients with depression were single/divorced compared with CLBP patients without depression (44.3 % vs 30.1 %). However, differences in marital status and sleeping difficulties were consistent with differences observed in major depression disorder [27].

Epidemiological, cross-sectional, and prospective studies suggest that insomnia, chronic pain and depression are a cluster of symptoms that are mutually interactive. Studies using a variety of methods, including neuroimaging, suggest the mesolimbic dopamine system has been proposed as a key factor in promoting the comorbidity of this cluster of symptoms, [32] and our observations of both higher ratings of pain severity as well as greater prevalence of sleep difficulties among CLBP patients with depression are additional supportive evidence to this body of data.

The adjusted mean HRQoL scores in the CLBP depression group were lower than in the CLBP group without depression. The health status using both Japanese norm-based as well was international scores, indicated significantly poorer outcomes for CLBP patients with depression compared with CLBP patients without depression. Lower PCS scores in CLBP patients with depression are indicative that a decline of mental health could have an effect on physical health in CLBP patients. A similar relationship has been reported among CLBP patients in the United Kingdom, in whom depression as measured by the Hospital Anxiety and Depression Scale (HADS) was correlated with PCS scores [33]. Labor force and absenteeism did not differ by depression status, potentially because of Japanese working habits, where there is a tendency for less sick leave claims compared to other countries [34]. However, presenteeism, overall work and activity impairment were lower in CLBP patients with depression, demonstrating that, even though employees are present at work, they are less productive than those CLBP patients without depression. Additional analyses indicated that presenteeism was closely related to overall work impairment. The current study also demonstrates more frequent use of healthcare among CLBP patients who have depression, consistent with the relationship between depression and healthcare visits recently demonstrated in the US using National Health and Nutrition Examination Survey data [35].

Treatment approaches, especially for Japanese workers, have focused on ergonomic approaches in the management of LBP. Consistent with a focus on musculoskeletal symptoms the majority of patients surveyed in our study were diagnosed with LBP by an orthopedist. However, recent studies highlight the importance of psychosocial risk factors in the development of CLBP [22, 23] and our data further highlights the need for mental health evaluation and treatment in addition to physical assessment and therapy.

One limitation of our study is that the analysis was cross-sectional. Therefore our results cannot indicate whether increased pain leads to depression, or whether depression leads to increased pain. Another limitation is selection bias that may not result in an all-encompassing representation of all patients with CLBP. The data were derived from opt-in surveys completed over the Internet. Compared to the general population our study population could be over-representative of individuals who live in urban environments and are technology literate.

Nakamura et al. has shown that chronic musculoskeletal pain does not necessarily improve with treatment and that patients have a high degree of dissatisfaction with it [11, 12]. Ineffective treatment may lead to “doctor shopping”. In our study, a significantly higher number of CLBP patients with depression than those without depression were using prescription pain medication (55.7 % vs 35.8 %, *P* = 0.002) indicating that depressed CLBP patients not only suffer more but may also find treatment less effective. Moreover, increased mental and physical suffering often require assistance. All these factors pose undue strain and increase societal cost.

## Conclusion

We have demonstrated that depression among CLBP patients is associated with higher pain scores and lower HRQoL scores, as well as lower labor productivity and increased healthcare use. Our results underscore the need to screen for depression in CLBP patients as an essential part of CLBP patient care.

## Abbreviations

BMI: Body mass index
BP: Bodily pain
CCI: Charlson Comorbidity Index
CLBP: Chronic low back pain
ER: Emergency room
GH: General health
HCP: Healthcare provider
HRQoL: Health-related quality of life
LBP: Low back pain
MCS: Mental component summary
MH: Mental health
NHWS: National Health and Wellness Survey
NRS: Numeric rating scale
NSAIDs: Non-steroidal anti-inflammatory drugs
OTC: Over-the-counter
PCS: Physical component summary
PF: Physical functioning
PHQ-9: Patient Health Questionnaire
RE: Role emotional
RP: Role physical
SF: Social functioning
SF-36v2: Medical Outcomes Study 36-Item Short Form Survey Instrument
SF-6D: Short form 6D
VT: Vitality
VTA: Ventral tegmental area
WPAI: Work Productivity and Activity Impairment
YLD: Years lived with disability
